# Palladium-Mediated Dealkylation of *N*-Propargyl-Floxuridine as a Bioorthogonal Oxygen-Independent Prodrug Strategy

**DOI:** 10.1038/srep09329

**Published:** 2015-03-19

**Authors:** Jason T. Weiss, Neil O. Carragher, Asier Unciti-Broceta

**Affiliations:** 1 Edinburgh Cancer Research UK Centre, MRC Institute of Genetics and Molecular Medicine, University of Edinburgh, Crewe Road South, Edinburgh EH4 2XR, UK

## Abstract

Herein we report the development and biological screening of a bioorthogonal palladium-labile prodrug of the nucleoside analogue floxuridine, a potent antineoplastic drug used in the clinic to treat advanced cancers. *N*-propargylation of the N3 position of its uracil ring resulted in a vast reduction of its biological activity (~6,250-fold). Cytotoxic properties were bioorthogonally rescued in cancer cell culture by heterogeneous palladium chemistry both in normoxia and hypoxia. Within the same environment, the reported chemo-reversible prodrug exhibited up to 1,450-fold difference of cytotoxicity whether it was in the absence or presence of the extracellular palladium source, underlining the precise modulation of bioactivity enabled by this bioorthogonally-activated prodrug strategy.

Bioorthogonally-activated prodrug therapies are a heterogeneous group of experimentally and clinically-used therapeutic strategies that are founded on a common principle: the site-specific activation of pharmaceutical substances by the mediation of non-biological, non-perturbing physical or chemical stimuli. While the nature and properties of the triggering stimulus can be manifestly diverse and seemingly unrelated (e.g. benign electromagnetic radiations^1^,^2^,^3^,^4^, metal-free click chemistry^5^,^6^,^7^, mild hyperthermia^8^,^9^, bioorthogonal organometallic (BOOM) reactions^10^,^11^,^12^,^13^, etc.), all these strategies are intrinsically linked by the wide-ranging notion of bioorthogonality coined by Bertozzi a decade ago^14^,^15^,^16^. By virtue of the bioorthogonal action of an external or internal source, precursors of various therapeutic substances, such as reactive oxygen species (photodynamic therapy^1^), cytotoxic small molecules (activated by photolysis^2^,^3^,^4^, or chemolysis^5^,^6^,^7^,^10^,^11^,^12^,^13^) or thermoresponsive drugs^8^,^9^, can be selectively activated within an anatomical area of a patient (e.g. a tumour), thus reducing the systemic adverse effects of the therapy.

Contributing to the “explosive” emergence of palladium in chemical biology^17^,^18^,^19^,^20^,^21^,^22^,^23^,^24^,^25^, we have recently reported a novel application of BOOM chemistry whereby polymer-entrapped palladium nanoparticles are deployed as extracellular heterogeneous catalysts in cancer cell culture to cleave protecting groups used to inactivate cytotoxic agents, thus restoring the drugs' pharmacological properties *in situ*^10^,^11^,^12^. Unlike other classes of locally-activated chemotherapies where the activating source generates a short-lived triggering stimulus, the catalytic nature of the reported BOOM reactions means that palladium-functionalized inserts could induce successive activating stimuli (catalytic cycles) in a continuous manner. Thereby, following the intratumoral implantation of a palladium-functionalized device (e.g. by minor surgery), cytotoxic drugs could be locally generated in the area surrounding the insert at levels sustained by the controlled flow (*via* dosing regulation) of a systemically-administered prodrug.

To develop this experimental approach into an effective therapeutic option, such novel class of drug precursors —i.e. palladium-labile prodrugs— have to be specifically designed to accomplish three goals: (i) eliminating their pharmacological properties; (ii) minimizing their susceptibility to enzymatic cleavage; and (iii) rendering them “cleavable” by palladium catalysis within physiological and pathophysiological environs. So far, only two prodrugs meeting such requirements have been described: 5-fluoro-1-propargyluracil (Pro-5FU, **1**)^10^, which generates cytotoxic 5-fluorouracil (5FU) upon palladium-mediated *N*-dealkylation; and *N*-propargyloxycarbonyl (*N*-Poc) gemcitabine (**2**)^11^ which undergoes rapid carbamate cleavage by Pd^0^ catalysis (Fig. 1a). On the basis of its high sensitivity to palladium and remarkable bioorthogonality (>500-fold less cytotoxic than 5FU), Pro-5FU (**1**) ideally features the sought-after properties required to implement a palladium-activated prodrug approach. However, since 5FU is a cytotoxic nucleobase of relatively low antiproliferative potency^10^,^12^, the levels of drug required to induce a strong phenotypic response could become a limiting factor for its clinical application. On the contrary, the BOOM activation of *N*-Poc-gemcitabine (**2**) results in the rapid and efficient generation of a potent anticancer drug (EC_50_ < 100 nM in tumoral cell lines)^11^. The caveat for this strategy is that, although it enables the reduction of the prodrug's bioactivity to levels that were satisfactory for cell culture studies (~25-fold decrease of cytotoxicity relative to the parental drug), the limitations in bioorthogonality of the *N*-Poc masking group were exposed *in vivo* in zebrafish embryos, where *N*-Poc-protected rhodamine showed low biochemical stability in the intestinal tract^11^. Hence, this masking strategy is likely to be suboptimal to satisfy the stability demands required for the translation of this chemistry into the clinic; particularly when the preferable route of administration is enteral.

Given the superior bioorthogonal properties of *N*-alkyl protecting groups and the efficacy of palladium to cleave propargyl groups at endocyclic nitrogen atoms with lactam/lactim tautomery^10^,^12^, we were prompted to investigate whether this novel chemistry could be compatible with drugs of higher structural complexity such as nucleoside analogues, which are reported to be significantly more potent than cytotoxic nucleobases^26^,^27^. Based on its chemical structure (Fig. 1b), we reasoned that the clinically-used anticancer drug floxuridine (also known as FUdR, **3**) was optimal for developing, and further validating, a propargylation/depropargylation strategy (coupling & decoupling chemistry^28^).

## Results and Discussion

FUdR is an antineoplastic antimetabolite that upon intracellular phosphorylation (on its 5′-OH) causes the inhibition of thymidylate synthetase, resulting in the disruption of DNA synthesis and cytotoxicity. It is the deoxynucleoside analogue of 5FU, possessing superior activity both in cancer cell lines and animal tumour model systems^29^. While 5FU is predominantly converted into its uridine analogue and incorporated into the RNA, the active forms of FUdR (phosphorylated derivatives) directly disrupt DNA replication, what is supposed to account for the comparative difference in cytotoxicity between the two antimetabolites^27^,^29^. FUdR is most commonly administered to patients with advanced colorectal, kidney and stomach cancer, including use as a specific treatment for patients to whom the primary colorectal tumour has metastasized to the liver, where it cannot be removed by surgery^30^. FUdR clinical trials for the treatment of other late-stage cancers, e.g. advanced pancreatic cancer^31^, have also found improved survival rates as compared to other chemotherapeutic agents. Treatment with FUdR is however limited by several severe side effects including dose limiting toxicities upon diarrhoea and neutropenia^30^,^31^. Apart from systemic adverse effects, its therapeutic action is limited by a short half-life^30^. FUdR is systemically catabolized into 5FU^29^,^32^, thus largely reducing the pharmacodynamic advantage of using FUdR over 5FU.

**Figure 2:**
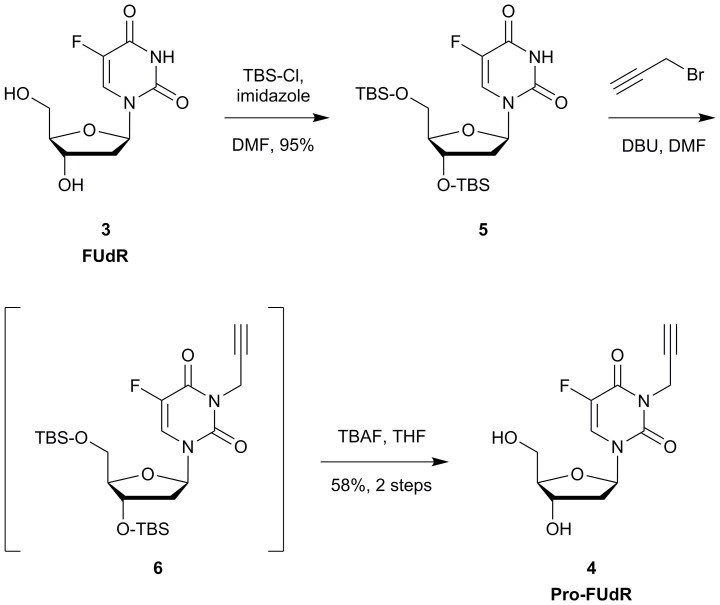
Semisynthesis of 3-propargylfloxuridine, 4 (Pro-FUdR).

To overcome FUdR pharmacokinetics issues, a number of prodrugs have been reported in the literature during the last decade^33^,^34^,^35^,^36^,^37^,^38^,^39^, among which include studies from Nishimoto, Tanabe and coworkers^36^,^37^,^38^,^39^, who have intensively investigated the development of a varied range of *N*3-modified stimuli-sensitive FUdR prodrugs. These masking strategies significantly decrease the cytotoxic effect of the drug, thus potentially improving FUdR's therapeutic window, but at levels similar to those found by us when using the carbamate masking strategy^11^. In contrast, a recent study reported by our group have shown that alkylation of the *N*3 position of the related drug 5FU results in superior suppression of drug's antiproliferative properties (>100-fold difference)^12^. Along with this fact, this hydrogen donor group is known to display a fundamental role in the substrate recognition of FUdR by both anabolic and catabolic enzymes^40^. Consequently, the propargylation of the *N*3 position of FUdR would not only suppress drug's pharmacological activity but should also protect it from its systemic metabolization into 5FU before reaching the target. Encouraged by this rationale and the unique chemistry of the *N*3 position (it possesses lactam/lactim tautomery), we decided to investigate the implementation of a bioorthogonal control of drug's pharmacodynamics via heterogeneous palladium chemistry. Pro-FUdR (**4**) was therefore synthesized following the 3 step procedure described in Fig. 2. In short, hydroxyl groups in the positions 3′ and 5′ of FUdR (**3**) were first protected using tert-butyldimethylsilyl chloride (TBS-Cl) and imidazole to yield bis-silylated derivative **5**. *N*-alkylation using propargyl bromide^41^ in the presence of 1,8-diazabicycloundec-7-ene (DBU) and subsequent desilylation of (non-isolated) intermediate **6** with tetrabutylammonium fluoride (TBAF) in THF yielded Pro-FUdR (**4**) in good overall yield.

**Figure 3:**
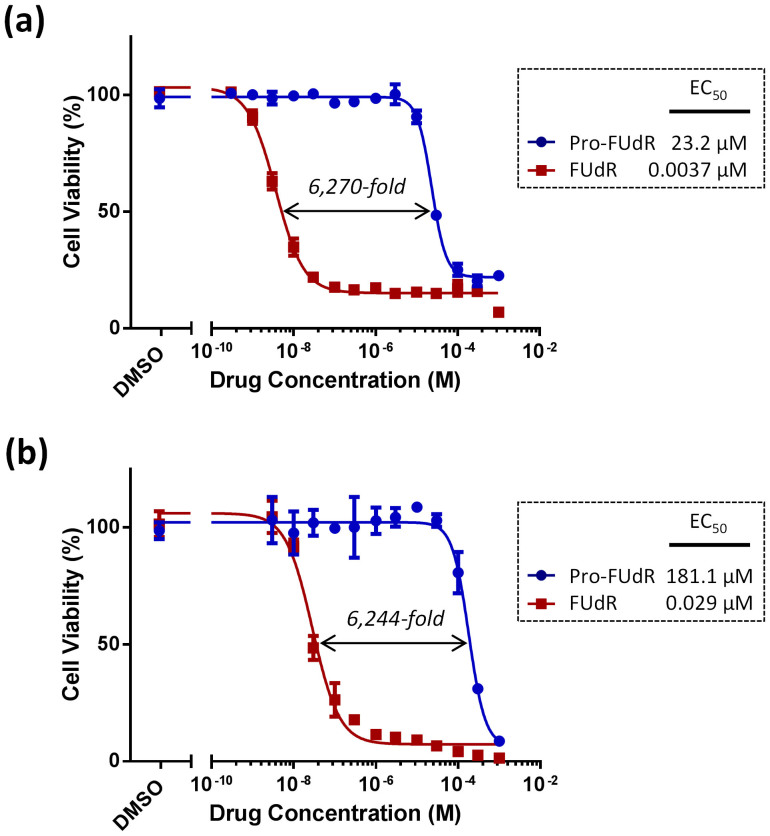
Semi Log dose response curves and calculated EC50 values of Pro-FUdR (in blue) in comparison to unmodified FUdR (in red) in (a) BxPC-3 and (b) HCT116 cells. Cell viability was determined at day 5 using PrestoBlue™ reagent and a microplate reader. Error bars: ±SD from n = 3.

The efficacy and stability of the deactivation strategy was first tested in cell culture by performing dose response studies with FUdR and Pro-FUdR in two human cancer cell lines: colorectal cancer HCT116 cells and pancreas adenocarcinoma BxPC-3 cells. As shown in Fig. 3, the 3-propargyl derivative of FUdR displayed a vast reduction in its biological properties relative to the parental drug, with a difference in antiproliferative activity between the two of >6,000-fold (EC_50_ (Pro-FUdR)/EC_50_ (FUdR)). This dramatic decrease in cytotoxicity does not only underline the relevant role played by the *N*3 position in the drug's biological properties, but also the remarkable stability ( = bioorthogonality) of the *N*-propargyl group to the cell metabolism.

Based on the validated biocompatibility of the components of the solid support^42^,^43^ and its suitable size (spheres of 150 μm in diameter, much larger than human cells), Pd^0^-resins consisting of palladium nanoparticles captured in PEG-grafted polystyrene particles (Supplemental Figure 1) were used to mediate the BOOM conversion of Pro-FUdR into FUdR. Since advanced solid tumors are estimated to have a slightly acidic pH (approx. 0.5–1.0 units below that of healthy tissues)^44^,^45^,^46^, *N*-dealkylation of Pro-FUdR's was tested in PBS (isotonic buffered solution) at two different pH's (6.5 and 7.5) to assess the compatibility of the reaction in both slightly alkaline and acidic pH's typical from normal and cancer-like extracellular environs. Pd^0^-resins (0.67 mg/ml) and Pro-FUdR (30 μM) were dispersed in the corresponding biocompatible solution and incubated at 37°C in a thermomixer. Reaction crudes were analyzed at 3 time points (4, 8 and 24 h) by HPLC using a UV detector. Chromatogram analysis showed that, while Pro-FUdR was fully stable in the absence of the triggering stimulus, it completely disappeared in the presence of Pd^0^ at each of the pH's tested before 24 h incubation, generating FUdR as the main reaction product (see HPLC chromatograms in Supplemental Figure 2). The effect of the pH was noticeable in the shorter incubation periods (4 and 8 h), with slightly superior reaction kinetics being observed at pH = 7.5 (see Table 1). This experiment suggests that the palladium-mediated *N*-dealkylation of Pro-FUdR is compatible with the range of pH expected to be found in both early (normoxic) and late-stage (hypoxic) cancers.

**Figure 4:**
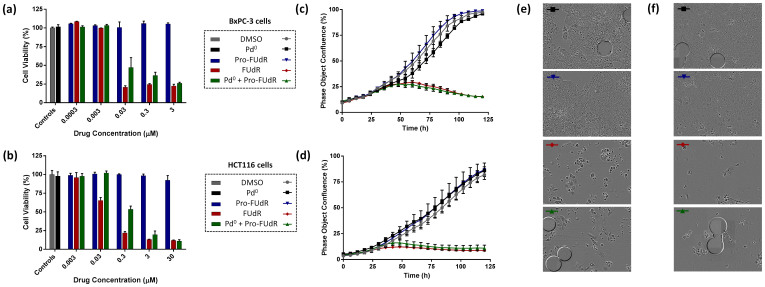
Palladium-mediated activation of Pro-FUdR in cancer cell culture: (a, c, e) BxPC-3 and (b, d, f) HCT116 cells. (a, b) Log dose response study of Pro-FUdR toxigenicity. Treatments: untreated cells (negative control); Pd^0^-resins (0.67 mg/mL, negative control); 0.0003–30 μM of Pro-FUdR (negative control); 0.0003–30 μM of FUdR (positive control); and Pd^0^-resin (0.67 mg/mL) + 0.0003–30 μM of Pro-FUdR (BOOM activation assay). All experiments, including the untreated cells, contained 0.1% *v*/*v* of DMSO. Cell viability was determined at day 5 of treatment using the PrestoBlue™ reagent (Life Technologies). Error bars: ±SD from n = 3. (c, d) Time-lapse imaging of cell proliferation: study of BOOM activation kinetics. Cell growth was monitored for 120 h using an IncuCyte ZOOM system in an incubator (5% CO_2_ and 37°C). [Drug/prodrug] = (c) 3 or (d) 10 μM. [Pd^0^-resins] = 0.67 mg/mL. Error bars: ±SD from n = 3. (e, f) Phase-contrast images of HCT116 (e) and BxPC-3 cells (f) after 5 days of treatment. Experiments are indicated with colored bars (top left corner), corresponding to each specific treatment displayed in (c, d). Pd^0^-resins are identified as grey spheres of approx. 150 μm in average diameter.

The efficacy by which the floxuridine prodrug is reverted to its active form via heterogeneous palladium catalysis ( = Pro-FUdR's toxigenicity^11^) was investigated in cancer cell culture. BxPC-3 and HCT116 cells were treated with Pd^0^-resins (0.67 mg/ml) and Pro-FUdR (0.0003 to 30 μM) separately (negative controls) or in combination (BOOM activation assay), and unmodified FUdR (0.0003–30 μM) used as the positive control. As expected, cells independently treated with either Pro-FUdR or Pd^0^-resins did not exhibit any reduction in cell viability (Fig. 4a, b). By contrast, the Pro-FUdR/Pd^0^-resins combinations displayed a strong toxigenic effect, evidence of the *in situ* generation of FUdR. Under the action of the triggering stimulus (Pd^0^-resins), Pro-FUdR exhibited an EC_50_ value of 0.319 μM in HCT116 cells (see Supplemental Figure 3). Considering that its EC_50_ without Pd^0^-resins was 181.1 μM, Pro-FUdR displayed a difference in biological properties of >560-fold from being in the presence or the absence of palladium (EC_50_ (Pro-FUdR)/EC_50_ (Pro-FUdR + Pd^0^)). In agreement with the lower levels of drug required to generate a cytotoxic phenotype in BxPC-3 cells, the EC_50_ value of the Pro-FUdR/Pd^0^-resins combination was 0.016 μM (Supplemental Figure 2), thus showing an even higher disparity between the inactive and reactivated drug (23.2 μM vs. 0.016 μM = 1,450-fold). To the best of our knowledge, Pro-FUdR exhibits one of the greatest therapeutic windows displayed *in vitro* by an antimetabolite prodrug, while having the smallest pro-moiety (38 atomic mass units) used to mask the activity of this family of drugs.

To evaluate whether the reaction kinetics between Pro-FUdR and Pd^0^-resins could match the direct cytotoxic effect provided by treatment with unmodified FUdR, cell proliferation was monitored for five days by time-lapse imaging using an IncuCyte ZOOM device. As shown in Fig. 4c–f, cells incubated with either Pro-FUdR (in blue) or Pd^0^-resins (in black) showed a growth curve equivalent to that of untreated cells (in grey). Conversely, combination of Pro-FUdR with Pd^0^-resins displayed a cytotoxic effect (in green) identical to that of cells incubated with the parental drug (in red). Pro-FUdR's ability to generate an immediate phenotypic effect only when Pd^0^-resins have been deployed in the culture media (see Supplemental Movies 1 and 2) demonstrates the efficacy of the deactivation strategy, the rapid reaction kinetics of the palladium-mediated *N*-depropargylation process and the high cytotoxic activity of the released drug (FUdR is 50 to 100-fold more potent than 5FU)^10^.

**Figure 5:**
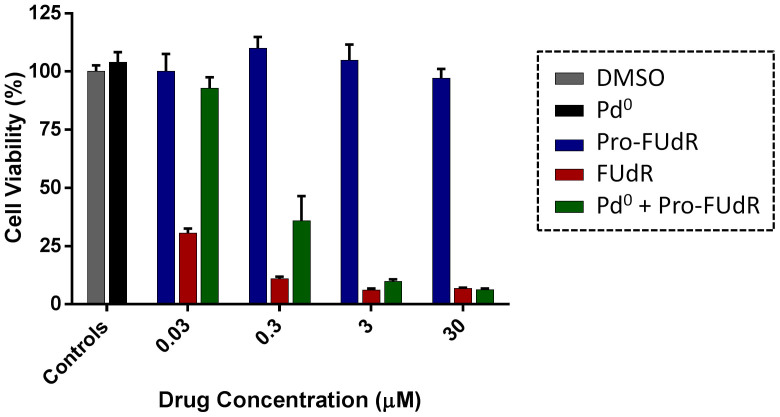
Palladium-mediated activation of Pro-FUdR in HCT116 cells under hypoxic conditions. Treatments: untreated cells (negative control); Pd^0^-resins (0.67 mg/mL, negative control); 0.03–30 μM of Pro-FUdR (negative control); 0.03–30 μM of FUdR (positive control); and Pd^0^-resin (0.67 mg/mL) + 0.03–30 μM of Pro-FUdR (BOOM activation assay). Cell viability was determined at day 5 using the PrestoBlue™ reagent (Life Technologies) and a microplate reader. Error bars: ±SD from n = 3.

Last, the compatibility of the Pro-FUdR's BOOM activation within oxygen deprived environs was investigated by performing the conversion assays with colorectal cancer HCT116 cells inside a hypoxic chamber ([O_2_] = 0.5%)^46^. As shown in Fig. 5, the toxigenic effect mediated by the Pro-FUdR/Pd^0^-resins combination in HCT116 cells in hypoxia were found to be equivalent to that of the combination in normoxic conditions (Fig. 4b), indicating that the oxygen levels have minimal or no influence on the BOOM reaction (see EC_50_ calculations in Supplemental Figure 4). While this result was anticipated based upon the mechanistic understanding of the dealkylation process^11^, it is nevertheless important because it suggests that the Pd^0^-mediated prodrug activation would be compatible with the anticipated oxygen deprived environment found in late-stage tumours. From a chemical point of view, given that the reductive environment of the hypoxic chamber is expected to significantly favour the oxidation state zero of the metal, these results further support that the dealkylation process is mediated by Pd^0^ in the liquid-solid interphase.

## Conclusions

In conclusion, a propargylation/depropargylation strategy has been successfully implemented to develop a truly-bioorthogonal palladium-labile prodrug of a nucleoside analogue, the cytotoxic agent floxuridine (FUdR): a potent drug employed in the clinic to fight advanced solid tumours. Propargylation of the NH group of FUdR's pyrimidine ring yielded a biochemically stable derivative (Pro-FUdR) displaying a vast reduction in cytotoxic activity relative to the unmodified drug (~6,250-fold). Complete palladium-mediated dealkylation of Pro-FUdR was shown to occur in less than 24 h across a range of pH from slightly acidic to physiological, allowing for the induction of a strong and rapid toxigenic effect in cancer cell culture regardless of the oxygen levels. This is the first study to report that palladium depropargylation chemistry is compatible with the relatively low pH and oxygen levels typically found in advanced human cancers. Within the same cellular environment, chemoreversible Pro-FUdR enabled an exquisite pharmacodynamic control by displaying a difference in biological activity of up to 1,450-fold whether it was in the presence or absence of palladium. From a synthetic perspective, the efficacy of palladium in triggering the dealkylation of a compound with the structural complexity of Pro-FUdR significantly expands the scope and applicability of the *N*-depropargylation approach as a bioorthogonal reaction. The remarkable biological and chemical properties of Pro-FUdR supports further investigations in clinically-relevant animal models and underline the precise modulation of prodrug activation that can be enabled by biocompatible palladium catalysis.

## Methods

### General

Cell lines were grown in culture media supplemented with serum (10% FBS) and L-glutamine (2 mM) and incubated in a tissue culture incubator at 37°C and 5% CO2. Human pancreas adenocarcinoma BxPC-3 cells (a kind gift from Dr Mark Duxbury) were cultured in Roswell Park Memorial Institute (RPMI) media. Human colorectal carcinoma HCT116 cells (a kind gift from Dr Van Schaeybroeck) was cultured in Dulbecco's Modified Eagle Media (DMEM).

### Cell viability studies

Cells were seeded in a 96 well plate format (at 1,000 cells/well for HCT116 and 2,500 cells/well for BxPC-3) and incubated for 48 h before treatment. Each well was then replaced with fresh media containing compound Pro-FUdR or FUdR and incubated for 5 days. Untreated cells were incubated with DMSO (0.1% *v*/*v*). Experiments were performed in triplicates. PrestoBlue™ cell viability reagent (10% *v*/*v*) was added to each well and the plate incubated for 1 h. Fluorescence emission was detected using a PerkinElmer EnVision 2101 multilabel reader (Perkin Elmer; excitation filter at 540 nm and emissions filter at 590 nm). All conditions were normalized to the untreated cells (100%) and curves fitted using GraphPad Prism using a sigmoidal variable slope curve.

### Pd^0^-mediated dealkylation of Pro-FUdR in cancer cell culture

HCT116 and BxPC-3 cells were plated as described above. Each well was then replaced with fresh media containing: Pd^0^-resins (0.67 mg/mL); Pro-FUdR (0.3 nM to 30 μM); FUdR (0.3 nM to 30 μM); or combination of 0.67 mg/mL of Pd^0^-resins + Pro-FUdR (0.3 nM to 30 μM). All experiments, including the untreated cells, contained 0.1% *v*/*v* of DMSO and were performed in triplicates. Cells were incubated with drugs for 5 days. PrestoBlue™ cell viability reagent (10% *v*/*v*) was added to each well and the plates were incubated for 1 h. Fluorescence emission was detected using a microplate reader and results normalized.

### Time-lapse IncuCyte proliferation study

Cell seeding density was optimized to reach confluency at day 5. HCT116 and BxPC-3 cells were plated as described above and each well was then replaced with fresh media containing: Pd^0^-resins (0.67 mg/mL); Pro-FUdR (3 μM for BxPC-3 and 10 µM for HCT116 cells); FUdR (3 or 10 μM); or combination of 0.67 mg/mL of Pd^0^-resins + Pro-FUdR (3 or 10 μM). All experiments, including the untreated cells, contained 0.1% *v*/*v* of DMSO and were performed in triplicate. Each well was imaged every 3 h over 5 d under standard incubation conditions using an IncuCyte™ ZOOM microscope placed inside an incubator. Image-based analysis of cell confluence was carried out using the IncuCyte™ software.

### Pd^0^-mediated dealkylation of Pro-FUdR in hypoxic model of colorectal cancer

Before treatment, 1,000 cells/well of HCT116 cells were seeded and incubated for 48 h under normoxic conditions. Cells were then treated as described above and immediately placed in a hypoxia chamber H35 Hypoxystation (Don Whitley, Yorks), which was flushed by a gas mixture calibrated to 0.5% O2 concentration. Cell viability experiments were performed and analysed at day 5 of treatment as described before.

## Author Contributions

J.T.W. prepared and characterized the materials, performed the experiments, analysed the data and wrote the methods section; N.O.C. supervised the biological assays and revised the manuscript; A.U.-B. designed the prodrug approach and the materials, planned and supervised the research, analysed the data and wrote the paper.

## Supplementary Material

Supplementary Information Supplemental Information

Supplementary Information Supplemental Movie 1

Supplementary Information Supplemental Movie 2

## Figures and Tables

**Figure 1:**
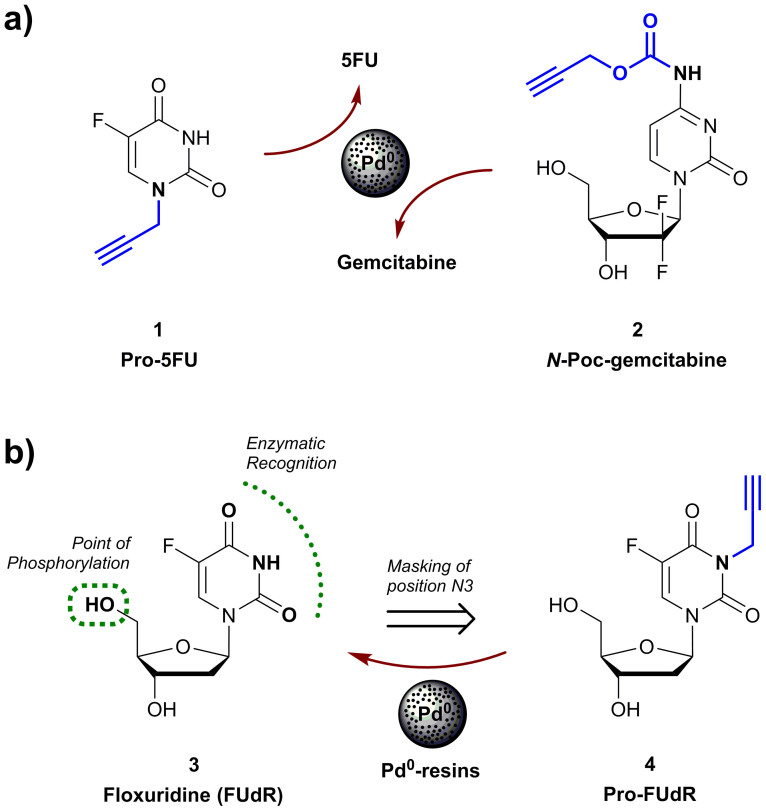
(a) Palladium labile precursors of 5FU (**1**) and gemcitabine (**2**). (b) FUdR (**3**) and the proposed palladium-labile precursor Pro-FUdR (**4**).

**Table 1:** Palladium-mediated conversion of Pro-FUdR into FUdR. Relative percentages as calculated by chromatogram peak integration

		REACTANTS			
		Pro-FUdR		Pro-FUdR + Pd^0^	
	Time	pH = 6.5	pH = 7.5	pH = 6.5	pH = 7.5
**PRODUCT (% of FUdR)**	**at t = 4 h**	N/D	N/D	26.6%	37.3%
	**at t = 8 h**	N/D	N/D	49.0%	65.9%
	**at t = 24 h**	0%	0%	100%	100%

## References

[b1] Castano A. P., Mroz P. & Hamblin M. R. Photodynamic therapy and anti-tumour immunity. Nat. Rev. Cancer 6, 535–545 (2006). 16794636 10.1038/nrc1894 PMC2933780

[b2] Bio M. *et al.* Site-specific and far-red light-activatable prodrug of combretastain A-4 using photo-unclick chemistry. J. Med. Chem. 56, 3936–3942 (2013). 23631389 10.1021/jm400139w

[b3] Tietze L. F., Müller M., Duefert S.-C., Schmuck K. & Schuberth I. Photoactivatable prodrugs of highly potent duocarmycin analogues for a selective cancer therapy. Chem. Eur J. 19, 1726–1731 (2013). 23225610 10.1002/chem.201202773

[b4] Velema W. A., Szymanski W. & Feringa B. L. Photopharmacology: beyond proof of principle. J. Am. Chem. Soc. 136, 2178–2191 (2014). 24456115 10.1021/ja413063e

[b5] Van Brakel R., Vulders R. C. M., Bokdam R. J., Grüll H. & Robillard M. S. A doxorubicin prodrug activated by the staudinger reaction. Bioconjugate Chem. 19, 714–718 (2008). 10.1021/bc700394s 18271515

[b6] Versteegen R. M., Rossin R., ten Hoeve W., Janssen H. M. & Robillard M. S. Click to Release: Instantaneous Doxorubicin Elimination upon Tetrazine Ligation. Angew. Chem. Int. Ed. Engl. 52, 14112–14116 (2013). 24281986 10.1002/anie.201305969

[b7] S. Murray B. S., Crot S., Siankevich S. & Dyson P. J. Potential of Cycloaddition Reactions To Generate Cytotoxic Metal Drugs In Vitro. Inorg. Chem. 53, 9315–9321 (2014). 25133591 10.1021/ic501438k

[b8] Clavel C. M. *et al.* Thermoresponsive Chlorambucil Derivatives for Tumour Targeting. Angew. Chem., Int. Ed. 50, 7124–7127 (2011). 10.1002/anie.201101133 21688360

[b9] Clavel C. M., Păunescu E., Nowak-Sliwinska P. & Dyson P. J. Thermoresponsive organometallic arene ruthenium complexes for tumour targeting. Chem. Sci. 5, 1097–1101 (2014).

[b10] Weiss J. T. *et al.* Extracellular palladium-catalysed dealkylation of 5-fluoro-1-propargyl-uracil as a bioorthogonally activated prodrug approach. Nat. Commun. 5, 3277 (2014). 24522696 10.1038/ncomms4277 PMC3929780

[b11] Weiss J. T. *et al.* Development and Bioorthogonal Activation of Palladium-Labile Prodrugs of Gemcitabine. J. Med. Chem. 57, 5395–5404 (2014). 24867590 10.1021/jm500531z PMC4078945

[b12] Weiss J. T. *et al.* *N*-alkynyl derivatives of 5-fluorouracil: susceptibility to palladium-mediated dealkylation and toxigenicity in cancer cell culture. Front. Chem. 2, 56 (2014). 25121087 10.3389/fchem.2014.00056 PMC4114543

[b13] Völker T., Dempwolff F., Graumann P. L. & Meggers E. Progress towards Bioorthogonal Catalysis with Organometallic Compounds. Angew. Chem. 53, 10536–10540 (2014). 25138780 10.1002/anie.201404547

[b14] Saxon E. & Bertozzi C. R. Cell surface engineering by a modified Staudinger reaction. Science 287, 2007–2010 (2000). 10720325 10.1126/science.287.5460.2007

[b15] Agard N. J., Prescher J. & Bertozzi C. R. A strain-promoted [3 + 2] azide-alkyne cycloaddition for covalent modification of biomolecules in living systems. J. Am. Chem. Soc. 126, 15046–15047 (2004). 15547999 10.1021/ja044996f

[b16] Sletten E. M. & Bertozzi C. R. Bioorthogonal chemistry: fishing for selectivity in a sea of functionality. Angew. Chem. 48, 6974–6998 (2009). 19714693 10.1002/anie.200900942 PMC2864149

[b17] Yusop R. M., Unciti-Broceta A., Johansson E. M. V., Sánchez-Martín R. M. & Bradley M. Palladium-mediated intracellular chemistry. Nat. Chem. 3, 239–243 (2011). 21336331 10.1038/nchem.981

[b18] Unciti-Broceta A., Johansson E. M. V., Yusop R. M., Sánchez-Martín R. M. & Bradley M. Synthesis of polystyrene microspheres and functionalization with Pd(0) nanoparticles to perform bioorthogonal organometallic chemistry in living cells. Nat. Protoc. 7, 1207–1218 (2012). 22653159 10.1038/nprot.2012.052

[b19] Li N., Lim R. K., Edwardraja S. & Lin Q. Copper-free Sonogashira cross-coupling for functionalization of alkyne-encoded proteins in aqueous medium and in bacterial cells. J. Am. Chem. Soc. 133, 15316–15319 (2011). 21899368 10.1021/ja2066913 PMC3184007

[b20] Spicer C. D., Triemer T. & Davis B. G. Palladium-mediated cell-surface labelling. J. Am. Chem. Soc. 134, 800–803 (2012). 22175226 10.1021/ja209352s

[b21] Michel B. W., Lippert A. R. & Chang C. J. A reaction-based fluorescent probe for selective imaging of carbon monoxide in living cells using a palladium-mediated carbonylation. J. Am. Chem. Soc. 134, 15668–15671 (2012). 22970765 10.1021/ja307017b

[b22] Spicer C. D. & Davis B. G. Rewriting the bacterial glycocalyx via Suzuki-Miyaura cross-coupling. Chem. Commun. 49, 2747–2749 (2013). 10.1039/c3cc38824g 23338477

[b23] Li J. *et al.* Ligand-free palladium-mediated site-specific protein labeling inside gram-negative bacterial pathogens. J. Am. Chem. Soc. 135, 7330–7338 (2013). 23641876 10.1021/ja402424j

[b24] Li J. *et al.* Palladium-triggered deprotection chemistry for protein activation in living cells. Nat. Chem. 6, 352–361 (2014). 24651204 10.1038/nchem.1887

[b25] Li N., Ramil C. P., Lim R. K. V. & Lin Q. A Genetically Encoded Alkyne Directs Palladium-Mediated Protein Labeling on Live Mammalian Cell Surface. ACS Chem. Biol. 10.1021/cb500649q. PMC4340352 25347611

[b26] Galmarini C. M., Mackey J. R. & Dumontet C. Nucleoside analogues and nucleobases in cancer treatment. Lancet Oncol. 3, 415–424 (2002). 12142171 10.1016/s1470-2045(02)00788-x

[b27] Galmarini C. M., Popowycz F. & Joseph B. Cytotoxic nucleoside analogues: different strategies to improve their clinical efficacy. Curr. Med. Chem. 15, 1072–1082 (2008). 18473803 10.2174/092986708784221449

[b28] Bielski R. & Witczak Z. Strategies for coupling molecular units if subsequent decoupling is required. Chem. Rev. 113, 2205–2224 (2013). 23153040 10.1021/cr200338q

[b29] van Laar J. A., Rustum Y. M., Ackland S. P., van Groeningen C. J. & Peters G. J. Comparison of 5-fluoro-2′-deoxyuridine with 5-fluorouracil and their role in the treatment of colorectal cancer. Eur. J. Cancer 34, 296–306 (1998). 9640213 10.1016/s0959-8049(97)00366-3

[b30] Power D. G. & Kemeny N. E. The role of floxuridine in metastatic liver disease. Mol. Cancer Ther. 8, 1015–1025 (2009). 19383854 10.1158/1535-7163.MCT-08-0709

[b31] Ardalan B. & Lima M. J. A phase II trial of FUdR in patients with advanced pancreatic cancer. Cancer Res. Clin. Oncol. 130, 561–566 (2004). 10.1007/s00432-004-0584-5 PMC12161780 15449185

[b32] Peters G. J., Laurensse E., Leyva A., Lankelma J. & Pinedo H. M. Sensitivity of human, murine, and rat cells to 5-fluorouracil and 5′-deoxy-5-fluorouridine in relation to drug-metabolizing enzymes. Cancer Res. 46, 20–28 (1986). 2415245

[b33] Tsume Y. *et al.* Enhanced absorption and growth inhibition with amino acid monoester prodrugs of floxuridine by targeting hPEPT1 transporters. Molecules 13, 1441–1454 (2008). 18719516 10.3390/molecules13071441 PMC6244841

[b34] Lorey M., Meiera C., De Clercq E. & Balzarini J. CycloSaligenyl-5-fluoro-2′ deoxyuridine monophosphate (cycloSal-FdUMP)-a new prodrug approach for FdUMP. Nucleosides Nucleotides 16, 789–792 (1997).

[b35] Tobias S. C. & Borch R. F. Synthesis and biological studies of novel nucleoside phosphoramidate prodrugs. J. Med. Chem. 44, 4475–4480 (2001). 11728193 10.1021/jm010337r

[b36] Tanabe K. *et al.* One-electron reduction characteristics of N(3)-substituted 5-fluorodeoxyuridines synthesized as radiation-activated prodrugs. Bioorg. Med. Chem. 11, 4551–4556 (2003). 14527551 10.1016/j.bmc.2003.08.001

[b37] Tanabe K., Makimura Y., Tachi Y., Imagawa-Sato A. & Nishimoto S. Hypoxia-selective activation of 5-fluorodeoxyuridine prodrug possessing indolequinone structure: radiolytic reduction and cytotoxicity characteristics. Bioorg. Med. Chem. Lett. 15, 2321–2324 (2005). 15837317 10.1016/j.bmcl.2005.03.013

[b38] Tanabe K., Ishizaki J., Ando Y., Ito T. & Nishimoto S. Reductive activation of 5-fluorodeoxyuridine prodrug possessing azide methyl group by hypoxic X-irradiation. Bioorg. Med. Chem. Lett. 22, 1682–1685 (2012). 22248856 10.1016/j.bmcl.2011.12.106

[b39] Tanabe K., Sugiura M., Ito T. & Nishimoto S. Synthesis and one-electron reduction characteristics of radiation-activated prodrugs possessing two 5-fluorodeoxyuridine units. Bioorg. Med. Chem. 20, 5164–5168 (2012). 22847019 10.1016/j.bmc.2012.07.008

[b40] Wittmann J. G. *et al.* Structures of the human orotidine-5′-monophosphate decarboxylase support a covalent mechanism and provide a framework for drug design. Structure 16, 82–92 (2008). 18184586 10.1016/j.str.2007.10.020

[b41] Sirivolu V. R., Chittepu P. & Seela F. DNA with branched internal side chains: synthesis of 5-tripropargylamine-dU and conjugation by an azide-alkyne double click reaction. Chembiochem 9, 2305–2316 (2008). 18780386 10.1002/cbic.200800313

[b42] Alcantar N. A., Aydil E. S. & Israelachvili J. N. Polyethylene glycol-coated biocompatible surfaces. J. Biomed. Mater. Res. 51, 343–351 (2000). 10880075 10.1002/1097-4636(20000905)51:3<343::aid-jbm7>3.0.co;2-d

[b43] Unciti-Broceta A., Díaz-Mochón J. J., Sánchez-Martín R. M. & Bradley M. The use of solid supports to generate nucleic acid carriers. Acc. Chem. Res. 45, 1140–1152 (2011). 22390230 10.1021/ar200263c

[b44] Tannock I. F. & Rotin D. Acid pH in tumors and its potential for therapeutic exploitation. Cancer Res. 49, 4373–4384 (1989). 2545340

[b45] Zhang X., Lin Y. & Gillies R. J. Tumor pH and its measurement. J. Nucl. Med. 51, 1167–1170 (2010). 20660380 10.2967/jnumed.109.068981 PMC4351768

[b46] Ward C. *et al.* New strategies for targeting the hypoxic tumour microenvironment in breast cancer. Cancer Treat. Rev. 39, 171 (2013). 23063837 10.1016/j.ctrv.2012.08.004

