# Supplementary material for: Palladium-Mediated Dealkylation of N-Propargyl-Floxuridine as a Bioorthogonal Oxygen-Independent Prodrug Strategy
Source: Sci Rep. 2015 Mar 19;5:9329. doi: 10.1038/srep09329 (PMC4365405; doi:10.1038/srep09329)
Supplement: Supplementary Information — Supplemental Information [file srep09329-s1.pdf]

Supplemental Information for

Palladium-Mediated Dealkylation of *N*-Propargyl-Floxuridine as a Bioorthogonal Oxygen-Independent Prodrug Strategy

J. T. Weiss, N. O. Carragher and A. Unciti-Broceta*

Edinburgh Cancer Research UK Centre, MRC Institute of Genetics and Molecular Medicine, University of Edinburgh, Crewe Road South, Edinburgh EH4 2XR, UK.

* To whom correspondence should be addressed. Email: Asier.Unciti-Broceta@igmm.ed.ac.uk

INDEX

1. General — **Pages 1**

2. Synthesis and characterization of novel compounds — **Pages 2-5**

- **2.1. Synthesis of 3'-*O*,5'-*O*-bis-TBS-5-fluoro-3-propargyldeoxyuridine (5)** — **Page 2**
- **2.2. ^{1}H- and ^{13}C-NMR of intermediate 5** — **Page 3**
- **2.3. Synthesis of 5-fluoro-1-propargyldeoxyuridine (Pro-FUdR, 4)** — **Page 4**
- **2.4. ^{1}H- and ^{13}C-NMR of prodrug 4** — **Page 5**

3. Synthesis of Pd^{0}-resins: palladium quantity and comments on catalysis — **Page 6**

4. Pd^{0}-mediated dealkylation of Pro-FUdR in PBS at different pH — **Page 7**

5. EC$_{50}$ of Pd^{0}-mediated dealkylation of Pro-FUdR in cancer cell culture — **Page 8**

6. EC$_{50}$ of Pd^{0}-mediated dealkylation of Pro-FUdR in hypoxic cell model of cancer — **Page 9**

1. General

Chemicals and solvents were purchased from Fisher Scientific, Sigma-Aldrich or VWR International Ltd. All reactions were performed at room temperature unless otherwise stated. NMR spectra were recorded at ambient temperature on a 500 MHz Bruker Avance III spectrometer. Chemical shifts are reported in parts per million (ppm) relative to the solvent peak. Rf values were determined on Merck TLC Silica gel 60 F254 plates under a 254 nm UV source. Purifications were carried out by flash column chromatography using commercially available silica gel (220-440 mesh, Sigma-Aldrich).

2. Synthetic and Characterization of Novel Compounds

2.1. Synthesis of 3'-*O*,5'-*O*-bis[(tert-butyl)dimethylsilyl]-5-fluoro-3-propargyldeoxyuridine (5).

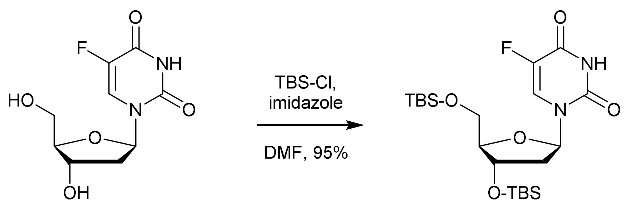

3 **5**

FUdR

Floxuridine **3** (300 mg, 1.22 mmol) and imidazole (606 mg, 8.90 mmol) were added to dry DMF (8 ml) and the resulting mixture stirred at for 5 min. Tert-butyldimethylsilyl chloride (TBS-Cl, 644 mg, 4.27 mmol) was then added to the mixture and the reaction stirred for 2 h. The solvents were removed *in vacuo*, re-dissolved in EtOAc (20 ml) and washed with ddH_2O (20 ml). The aqueous layer was then washed two more times with EtOAc, the organic layer was then washed with brine (60 ml) and dried over anhydrous $MgSO_4$. The product was then purified by column chromatography with 2:1 EtOAc/hexane to yield compound **5** as a white solid (552 mg, 95%). ^{1}H NMR (500 MHz, $CDCl_3$) δ 9.31 (d, *J* = 3.9, 1H), 8.04 (d, *J* = 6.3, 1H), 6.29 (td, *J* = 6.3, 1.6, 1H), 4.44 – 4.38 (m, 1H), 3.96 – 3.90 (m, 2H), 3.77 (t, *J* = 6.0, 1H), 2.32 (ddd, *J* = 13.3, 6.1, 3.8, 1H), 2.09 – 2.01 (m, 1H), 0.94 – 0.91 (m, 9H), 0.90 – 0.87 (m, 9H), 0.12 (t, *J* = 3.2, 6H), 0.07 (t, *J* = 2.8, 6H). ^{13}C NMR (126 MHz, $CDCl_3$) δ 157.05 (d, *J* = 26.9), 148.98, 140.63 (d, *J* = 236.6), 124.43 (d, *J* = 34.0, CH), 88.23 (CH), 85.67 (CH), 71.67 (CH), 62.82 (CH_2), 41.95 (CH_2), 26.02 (CH_3), 25.85 (CH_3), 18.55, 18.12, -4.61 (d, *J* = 31.1, CH_3), -5.45 (d, *J* = 3.2, CH_3). LC-MS (m/z): 475.4 $[M+H]^+$.

2.2. ^{1}H- and ^{13}C-NMR of intermediate 5.

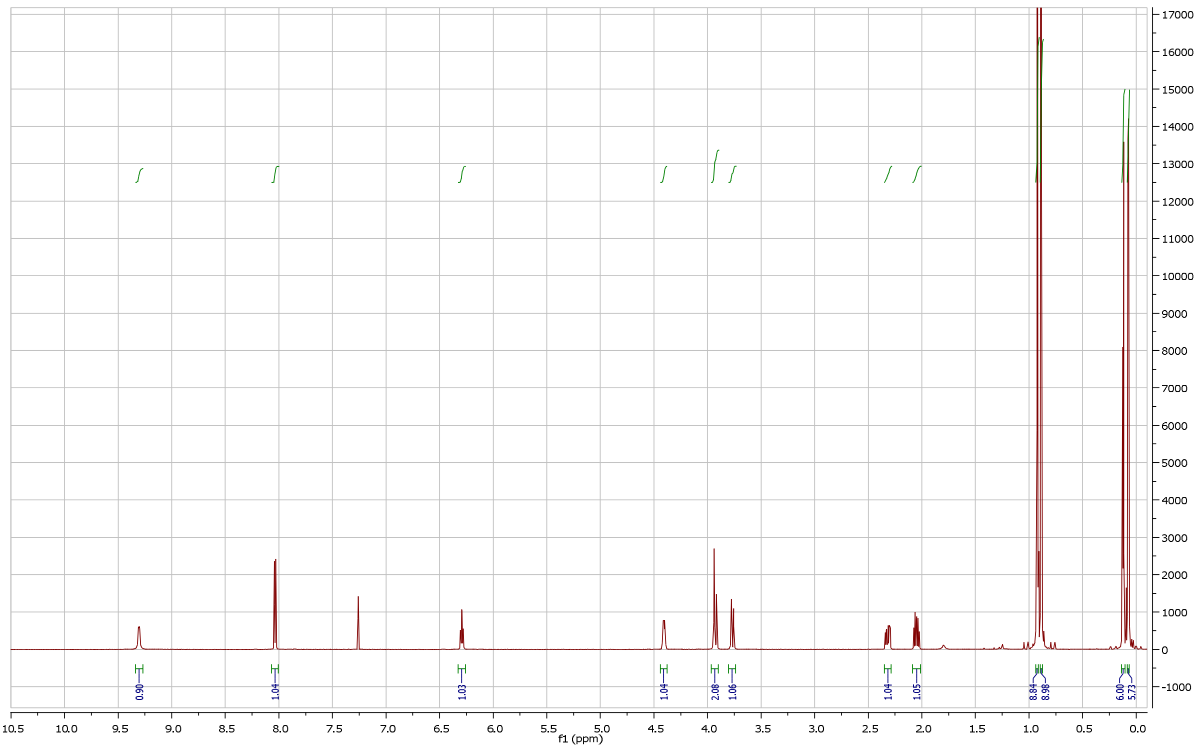

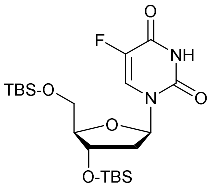

5

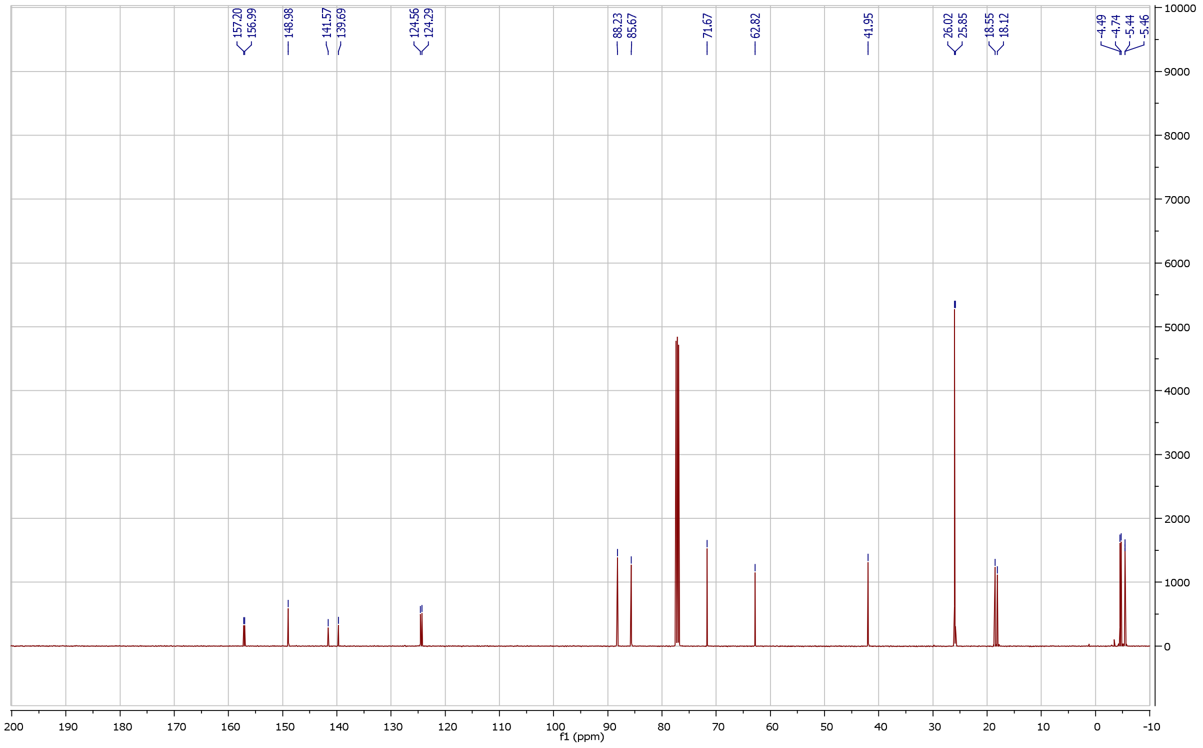

2.3. Synthesis of 5-fluoro-3-propargyldeoxyuridine (Pro-FUdR, 4).

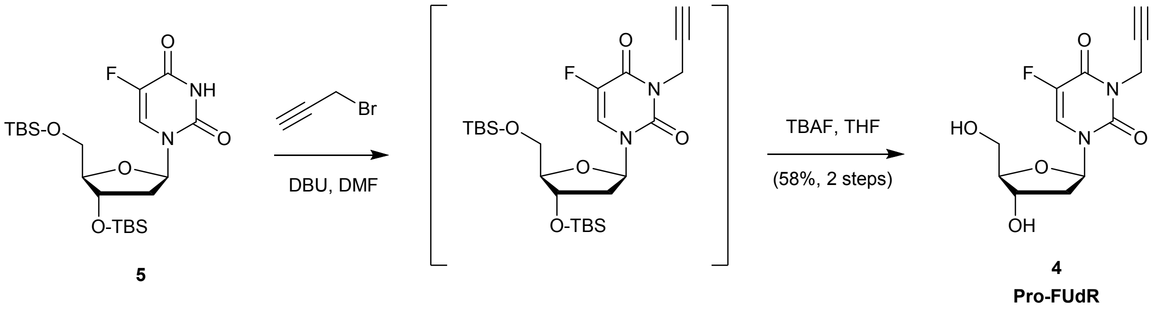

A solution of compound 5 (150 mg, 0.32 mmol) and DBU (168 µl, 1.12 mmol) in dry DCM (3 ml) was stirred for 5 min before adding dropwise propargyl bromide (120 µl, 0.93 mmol). The reaction was stirred for 30 min and monitored by TLC till completion. Afterwards, additional 17 ml of DCM was added and the mixture was washed once with ddH_2O (20 ml). The aqueous layer was then washed twice more with DCM (20 ml). The organic layers were collected, washed twice with brine (60 ml), dried over anhydrous $MgSO_4$ and concentrated in *vacuo.* Without further purification, the product was dissolved in a 1M TBAF solution in THF (2.5 ml) and stirred for 1 h. The mixture was concentrated *in vacuo*, dissolved in 25 % IPA in $CHCl_3$ (20 ml) and washed once with ddH_2O (20 ml). The aqueous layer was then washed twice more with 25 % IPA in $CHCl_3$ (20 ml). The organic layers were collected, dried over anhydrous $MgSO_4$ and the resulting crude purified by column chromatography using 6 % MeOH in DCM as eluent to yield compound **4** as a colourless sticky solid (52 mg, 58 %). ^{1}H NMR (500 MHz, DMSO) δ 8.36 (d, J = 7.0, 1H), 6.18 (td, J = 6.4, 1.5, 1H), 5.28 (d, J = 4.3, 1H), 5.19 (t, J = 4.9, 1H), 4.54 (t, J = 2.0, 2H), 4.25 (dt, J = 8.7, 4.3, 1H), 3.81 (q, J = 3.3, 1H), 3.61 (qdd, J = 11.9, 4.9, 3.5, 2H), 3.18 (t, J = 2.4, 1H), 2.19 – 2.12 (m, 2H). ^{13}C NMR (126 MHz, DMSO) δ 155.63 (d, J= 26.4), 148.28, 139.29 (d, J= 228.6), 124.04 (d, J = 34.6, CH), 87.72 (CH), 85.68 (CH), 78.41, 73.52 (CH), 69.93 (CH), 60.86 (CH_2), 39.94 (CH_2), 30.49 (CH_2). LC-MS (m/z): 319.0 $[M+Cl]^-$. HRMS (m/z): $[M +Cl]^-$ calcd for $C_{12}H_{13}O_5N_2F_1Cl_1$ 319.0497; found 319.0502. Purity: > 95% (analysed by HPLC).

2.4. ^{1}H- and ^{13}C-NMR of prodrug 4.

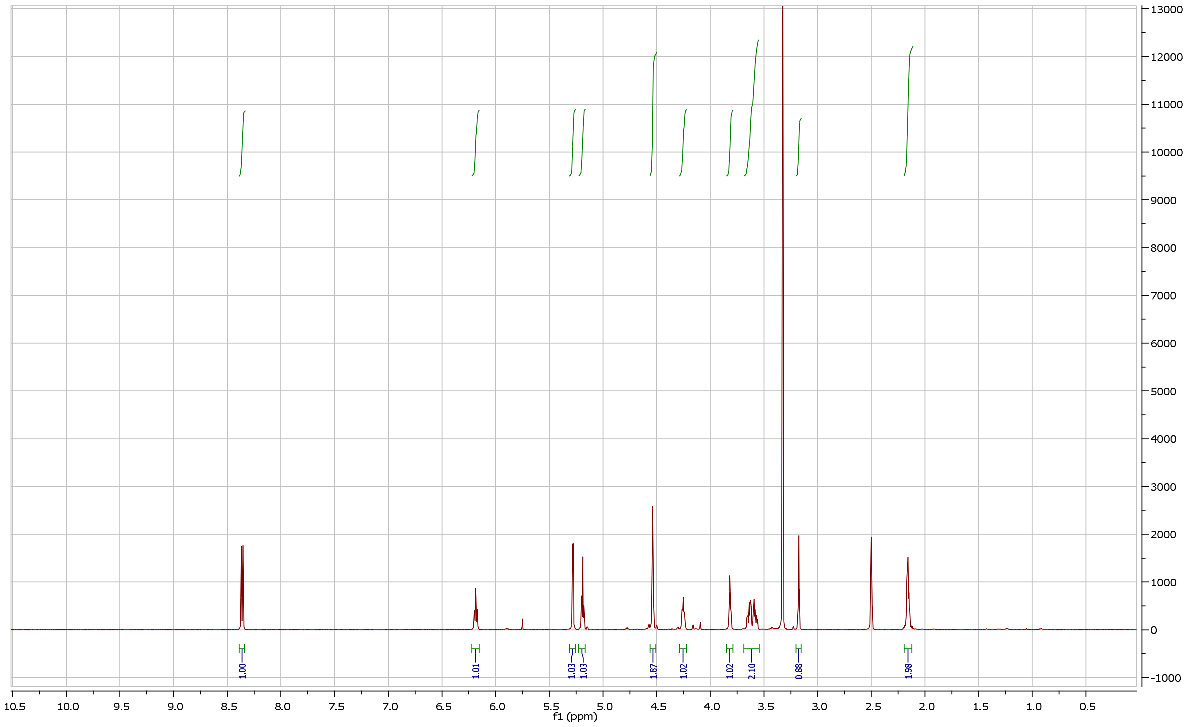

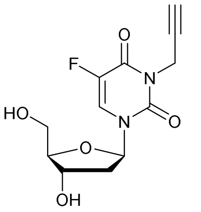

4

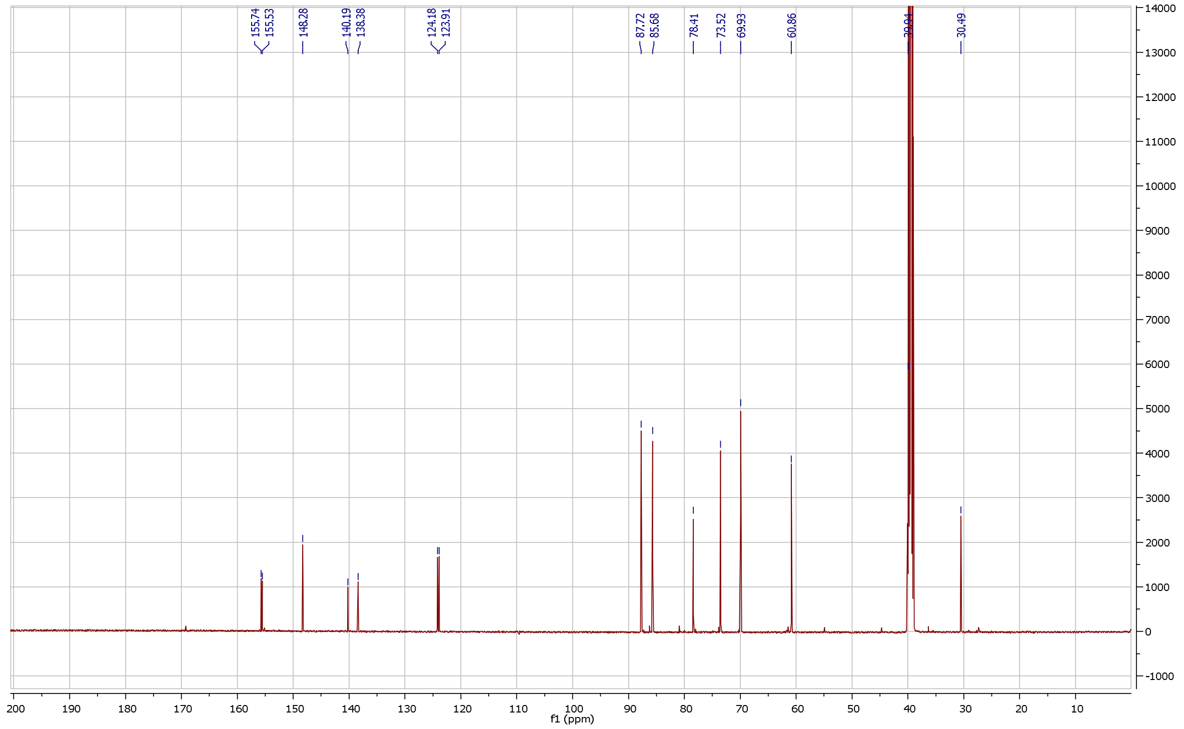

3. Synthesis of Pd^0-resins: characterization, palladium quantity and comments on catalysis.

Pd^0-functionalized resins were prepared from NovaSyn TG amino resin HL (0.39 mmol NH2 / g) as previously described [ref 1a, Weiss et al, Nat Commun]. The content of palladium in the Pd^0-resins was determined by inductively coupled plasma-optical emission spectrometry and found to be 4.4 % *w*/*w* in Pd. According to this quantification, a solution containing 0.67 mg/mL Pd^0-resins would contain a total concentration of [Pd^0]= 277 µM. Palladium-functionalized resins were also imaged by scanning electron microscopy (SEM) and transmission electron microscopy (TEM) (see Figure 1). As observed by TEM of a cross-section of a Pd^0-resin, palladium is found as dark nanoparticles of 5 nm regularly distributed across the resins. Considering that Pd^0-resins are spherical (Supp. Fig 1a,b) and compact polymeric structures, it is expected that, from the total amount of palladium nanoparticles distributed across the polymer network, only those at the surface of the spheres would become in direct contact with the liquid phase and with the chemicals (Pro-FUdR) dissolved in the medium. Consequently, the actual amount of palladium that would be involved in the heterogeneous catalytic process would be just a fraction (most certainly <20%) of the total amount of palladium "entrapped" in the resins.

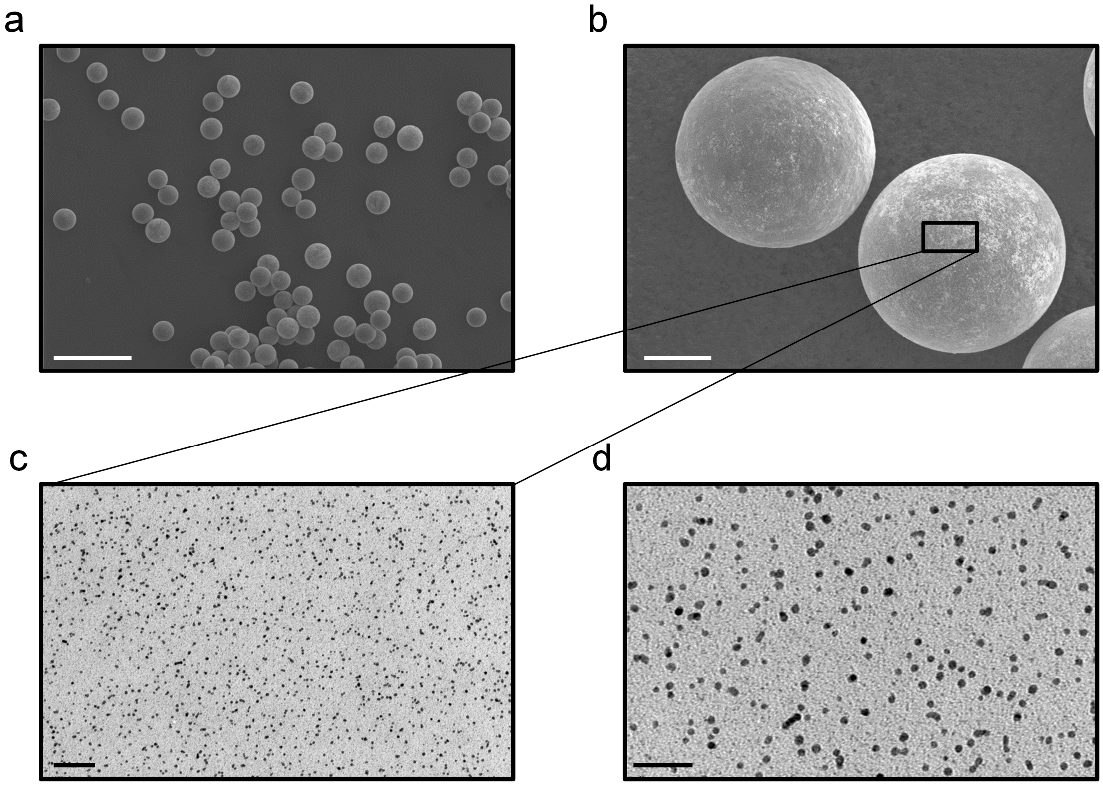

Supplemental Figure 1. SEM and TEM images of Pd^0 resin. (**a-b**) SEM images of Pd^0 resins. (**a**) Pd^0 resins at approximately 22 magnification (scale bar: 500 µm); (**b**) Pd^0 resins at approximately 180 magnification (scale bar: 50 µm) (**c-d**) TEM cross section of Pd^0 resin. (**c**) cross section at approximately 6 X 10^5 magnification (scale bar: 100 nm) (**d**) cross section at approximately 1.6 X 10^6 magnification (scale bar: 50 nm).

4. Pd^0-mediated dealkylation of Pro-FUdR in PBS at different pH.

The pH of PBS was adjusted with 1 N solutions of hydrochloric acid or sodium hydroxide using a pH meter (Mettler Toledo) to make isotonic solutions of pH 6.5 and 7.5. PBS solutions of Pro-FUdR (30 µM) and 0.67 mg/mL of Pd^0-resins (total volume= 1 mL) were shaken at 1,200 rpm and 37 °C in a Thermomixer. Reaction crudes were monitored at 4 h, 8 h and 24 h by analytical HPLC (Agilent) using the UV detector at 280 nm to avoid the detection of PBS salts. HPLC chromatograms are shown in Supplemental Figure 2.

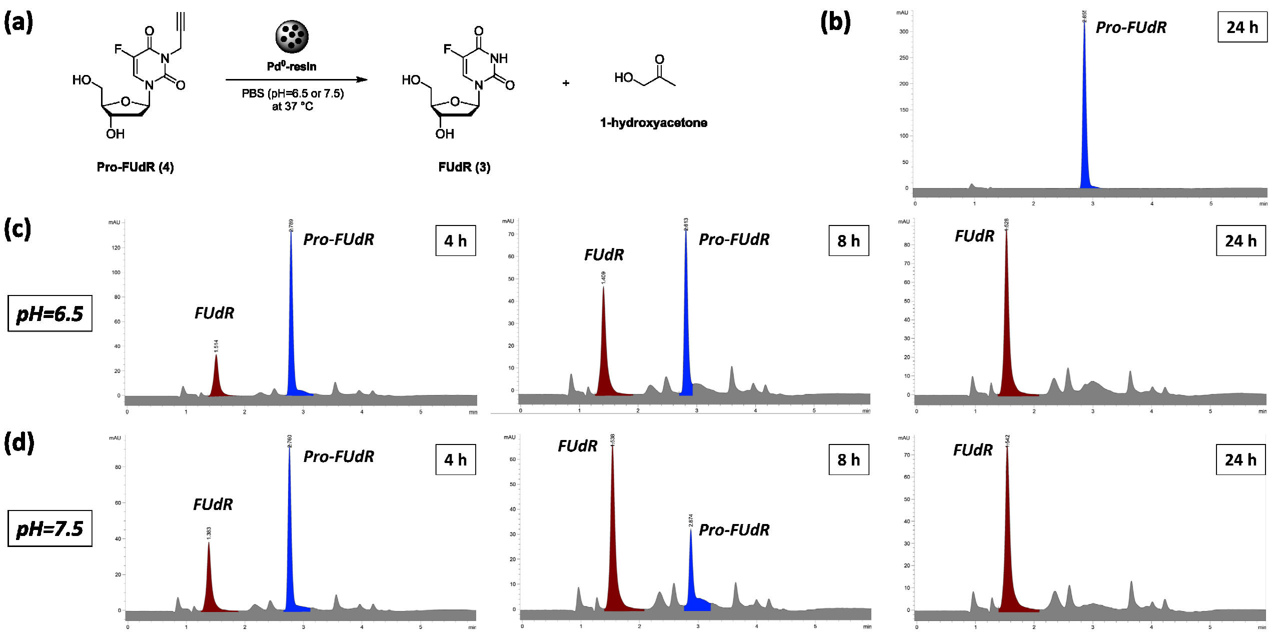

Supplemental Figure 2. (a) Palladium-mediated dealkylation of Pro-FUdR into FUdR. PBS solutions of Pro-FUdR (30 µM) were incubated with 0.67 mg/mL of Pd^0-resins at 37 °C for 24h and the crude reaction analyzed by HPLC (UV detector 280 nm). Eluent A: water and formic acid (0.1%); eluent B: acetonitrile, formic acid (0.1%); A/B = 95 : 5 to 5 : 95 in 3 min, isocratic 1 min, 5 : 95 to 95 : 5 in 1 min, isocratic 1 min. **(b)** Control experiment: HPLC chromatogram of Pro-FUdR after 24 h incubation at the same conditions but without palladium. **(c,d)** HPLC chromatograms of Pro-FUdR after 4 h (left panel), 8 h (middle panel) or 24 h (right panel) incubation with 0.67 mg/mL of Pd^0-resins at 37 °C in PBS adjusted to **(c)** pH= 6.5 or **(d)** pH= 7.5.

5. EC_{50} of Pd^0-mediated dealkylation of Pro-FUdR in cancer cell culture.

Dose response curves were plotted to determine EC_{50} values for the BOOM activation assay in comparison with the parental drug. Results are shown in Supplemental Figure 3.

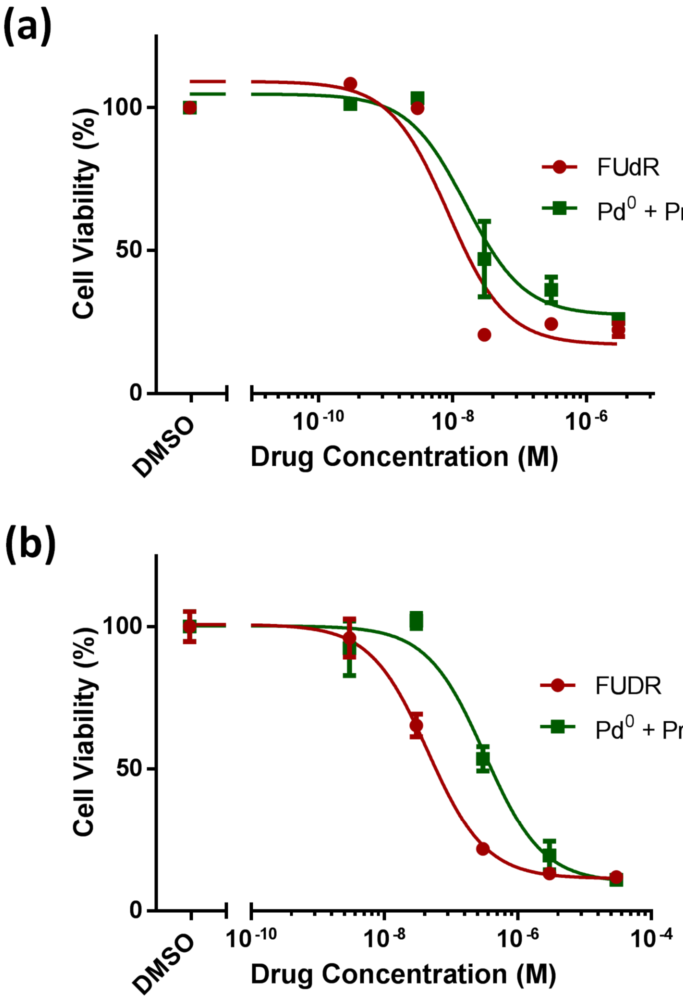

Supplemental Figure 3. Log dose response curves and calculated EC_{50} values of Pro-FUdR + Pd^0-resins combinations (in green) in comparison to unmodified FUdR (in red) in **(a)** BxPC-3 and **(b)** HCT116 cells. Cell viability was determined at day 5 using PrestoBlue™ reagent and a microplate reader. Error bars: ± SD from n=3.

6. EC_{50} of Pd^0-mediated dealkylation of Pro-FUdR in hypoxic cell model of colorectal cancer.

Plotting of dose response curves allowed determining the EC_{50} values for the BOOM activation assay in comparison with the parental drug. As shown in Supplemental Figure 4, the effect mediated by the prodrug in the presence of Pd^0-resins was in the range of 9-10 fold lower than the one mediated by the unmodified drug, similar toxigenic effect to the one obtained in normoxia.

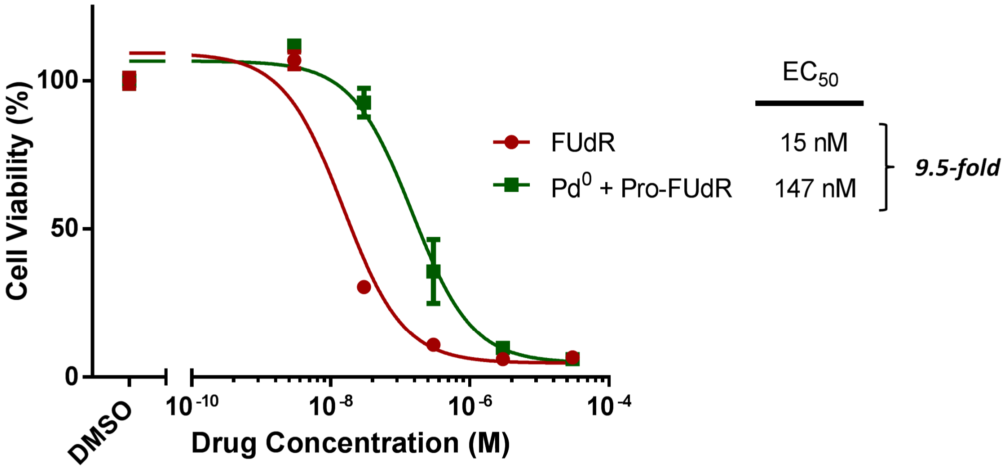

Supplemental Figure 4. Palladium-mediated activation of Pro-FUdR in HCT116 cells under hypoxic conditions. Log dose response curves and calculated EC_{50} values of Pro-FUdR + Pd^0-resins combinations (in green) in comparison to unmodified FUdR (in red) in HCT116 cells under hypoxic conditions. Cell viability was determined at day 5 using the PrestoBlue™ reagent (Life Technologies) and a microplate reader. Error bars: ± SD from n = 3.
